# User experiences on implementation of patient reported outcome measures (PROMs) in a Haematological outpatient clinic

**DOI:** 10.1186/s41687-020-00256-z

**Published:** 2020-10-28

**Authors:** Stine Thestrup Hansen, Mette Kjerholt, Sarah Friis Christensen, John Brodersen, Bibi Hølge-Hazelton

**Affiliations:** 1 Department of Haematology, Zealand University Hospital, Vestermarksvej 9, 1.sal, 4000 Roskilde, Denmark; 2 Department of Regional Health Research, Faculty of Health Sciences, University of Southern Denmark, Odense, Denmark; 3 Department of Plastic Surgery and Breast Surgery, Zealand University Hospital, Roskilde, Denmark; 4 Faculty of Health and Medical Sciences, University of Copenhagen, Copenhagen, Denmark; 5 Section of General Practice and Research Unit for General practice, Department of Public Health, Faculty of Health and Medical Sciences, University of Copenhagen, Copenhagen, Denmark; 6 Primary Health Care Research Unit, Zealand Region, Denmark; 7 The Research Support Unit, Zealand University Hospital, Roskilde, Denmark

**Keywords:** Patient reported outcome measures, Haematology, Qualitative, User experiences, Cancer, Consultations

## Abstract

**Background:**

PROMs can help healthcare professionals gain an improved understanding of patients’ physical burdens, functional levels, and (health-related) quality of life throughout disease and medical treatment. The aim of this study was to investigate the barriers and potential opportunities PROMs may present in a haematological outpatient clinic from three different perspectives: patients, nurses and haematologists.

**Methods:**

The present study synthesizes three previously published studies that separately explored the experiences of patients, nurses and haematologists when implementing PROMs. The studies were all guided by the qualitative methodology Interpretive Description, including a focused ethnographic approach, to develop implications for future practice.

**Results:**

The overall themes that emerged from the analysis were “Structural similarities influence the adoption of PROMs” and “Different perspectives on the potential of PROMs.”

**Conclusion:**

Across the different user groups in the haematological outpatient clinic, the use of PROMs was thwarted due to an unquestioned commitment to biomedical knowledge and the system’s rationality and norms: PROM data was not used in patient consultations. Nurses and haematologists expressed different preferences related to potential future PROMs and different objectives for PROMs in clinical practice. From the different perspectives of the patients, nurses and haematologists, PROMs were not compatible with clinical practice. Further research is recommended to develop PROMs validated for use in haematological outpatient clinics. Moreover, implementation strategies adjusted to the structural barriers of the system are crucial.

**Supplementary information:**

**Supplementary information** accompanies this paper at 10.1186/s41687-020-00256-z.

## Introduction

Implementation of Patient Reported Outcome Measures (PROMs) in clinical practice is recommended by patient organizations, politicians and researchers [1–5] to allow patients to report on personal experiences, for example treatment burden, treatment effects, functional levels and health-related quality of life. By integrating systematically-collected PROM assessments into clinical practice, the healthcare system can strengthen patient-professional relations by increasing patient involvement and the individualization of patient care trajectories [6, 7]. For healthcare professionals, PROMs are intended to be a supportive tool during patient visits, useful for clinical decision-making and communication [5, 8].

For patients diagnosed with haematological disorders such as chronic haematological neoplasms, person-centred care through partnership with haematologists and nurses is crucial to reduce potential disease burden [9, 10], including for example pain, pruritic conditions, or psychosocial challenges [11]. Person-centred care is defined as *“an approach to practice that is established through the formation and fostering of therapeutic relationships between all care providers, patients, and others significant to them. Person-centred care is underpinned by values of respect for persons, individual right to self-determination, mutual respect, and understanding”* [12]. PROMs are one way to support such relationships by assessing the nature and severity of the physical, psychosocial and functional disabilities [13, 14] which patients may encounter during and/or over time with a disease [10, 13]. The identification of such disabilities can lead to alleviation of the burdens caused by the illness or its treatment [4, 15]. Ultimately, the use of PROMs may help improve the quality of life for patients diagnosed with haematological disorders [4, 13, 16], who are a heterogeneous and challenging population. However, outcomes do not improve automatically simply because PROMs are applied in a clinical context. Successful use of PROMs requires a chain of cause and effect, including a) ensuring the PROM measures what it claims to measure [17]; b) use of the PROM instrument by healthcare professionals to intervene and respond appropriately to detected increases in symptom burden; and c) ensuring the PROM-instigated intervention has a positive effect on e.g. the patients’ health-related quality of life, which again should be measured using valid and reliable PROMs [18, 19].

Further research is needed to meet the increasing demand for implementation of PROMs, especially regarding the underlying assumption that PROMs will address the intended need. Significant problems arise when generalizing PROM findings from the population level to the individual level, or from one specialty to another, and PROM instruments must balance standardization and individualization, depending on the purpose of the measurement [20, 21]. Integration of PROMs in clinical practice without such considerations could potentially weaken the intended effect, introducing measure-related weaknesses. Measure-related weaknesses are rarely considered but can influence the results, due to: appropriateness of the outcomes; whether the instrument assesses the desired constructs; or relevance or focus of the scope [22]. Reports on the experiences of clinicians and the institutional implications of such perspectives are lacking [23], as are reports on patient experiences with PROMs [5]. Previous researchers have suggested that future PROM studies should investigate the experiences of clinicians, PROM experts, and patient representatives [4, 24]. Furthermore, researchers have made an effort to identify barriers and facilitators to PROM implementation, but have not achieved consensus, as local factors are highly influential, including choice of PROM instrument, implementation or usage policies, technological factors, knowledge, culture and context [5, 25–27].

Previously, three qualitative studies were published on three PROM user groups in an outpatient haematology clinic, focusing on (I) patients and their experiences with PROMs, (II) nurses’ experiences with PROMs, and (III) haematologists experiences with PROMs. The studies were performed as part of a larger multimethod project focused on one integrated clinical setting. The studies aimed to explore user perspectives when PROMs were introduced in clinical care of patients with chronic haematological cancer (see Additional file 1 and Additional file 2). A qualitative conceptual framework guided the study, using Interpretive Description^1^ with a focused ethnographic approach, including participant observations and interviews. Habermas’ social theory of communicative action^2^ inspired the analysis. A major finding examined in the three studies was that patients struggled to identify with the subject matter of the questions, and the questionnaires were associated with low content validity. Doctors and nurses rarely discussed PROMs with patients, a finding both directly observed in field studies and reported in patient interviews [30]. The nurses’ experiences were affected by their limited capacity to use PROM data during patient visits. Still, nurses believed that PROMs might have potential to support clinical practice by identifying new information about patient conditions and needs for supportive care [31]. The haematologist user group was either resistant to or supportive of PROMs implementation. No patients, nurses or haematologists were observed to refer to or use PROM data during consultations [32].

Examining all three of these user group perspectives, the theme emerged of fragmented practice, with diverse perspectives related to the implementation of PROMs. Still, similar patterns may be observed. The present synthesizing study was required to inform future practice for PROM implementation and to analyse what happens in clinical practice when PROMs are implemented. Based on the three individual qualitative studies of user experiences, this study aimed to investigate: *What are the barriers and potential opportunities PROMs may present in a clinical haematological outpatient clinic from all three user group perspectives?*

## Methods

### Design and participants

This qualitative study was part of a multimethod project [33] carried out at a large outpatient haematology clinic at a Danish university hospital from March 2017 to June 2020. The multimethod project included a randomized controlled trial (RCT) and a qualitative study (including four sub-studies, three of which were mentioned in the Introduction). The current and fourth study synthesizes the three sub-studies, all conducted by the same authors.

For the RCT, patients were included for a two-year period and randomized into three study groups:

- Group 1: Completed PROMs and PROM data was made available to nurses and haematologists in the electronic medical record system;
- Group 2: Completed PROMs but PROM data was not made available to nurses and haematologists;
- Group 3: A control group that followed standard departmental procedures; patients were not asked to complete a PROM.

Patient inclusion criteria for the RCT were:

- Newly diagnosed patients age > 18 years;
- Patients diagnosed with chronic haematological neoplasms (except multiple myeloma) who had been transferred into follow-up consultations, either directly or after completing an initial chemotherapy treatment.

Exclusion criteria were patients with physical and/or mental issues related to compliance and/or adherence.

For Group 1 patients, the authors hypothesized that the PROMs would be used by nurses and haematologists to initiate supportive care and rehabilitation. In Group 2, the authors hypothesized that patients would be prompted to reflect and discuss issues from the PROMs with doctors or nurses during consultations, potentially initiating supportive care and rehabilitation [34]. More details related to the multimethod project parameters have been reported elsewhere [30–32]. The three qualitative user group studies were guided by the inductive and constructive research methodology Interpretive Description (ID), using a focused ethnographic approach [28, 35]. Focusing on inquiry for the purposes of applied practice, ID is a flexible strategy allowing approaches to adapt to fit the nature of the purpose and inform practice-oriented research, and thus relevant for use in this study. A critical theory framework from the German sociologist and philosopher Jürgen Habermas inspired the interpretation and discussion of the findings, including Habermas’ concepts of system versus lifeworld, rationalities, intra-subjectivity, and norms [36, 37].

Triangulation was applied to synthesize data, identify similarities and differences, and to discuss how the three different perspectives may affect the implementation of PROMs in clinical practice, aiming to enhance data richness and develop a comprehensive understanding of phenomena [38]. The triangulation of data sources included triangulation of patient, nurse and haematologist perspectives; method triangulation was also applied to field observations and interviews [38].

The three qualitative user group studies included the following informants: 16 patients, 9 nurses and 14 haematologists, all of whom were purposefully selected for each sub-study [28]. Patient participants were recruited from the RCT study that was a part of the larger multimethod project; patients represented the three randomized groups within the RCT, to reflect the three different forms of consultations as well as patients’ gender and age. Nurses and haematologists were theoretically sampled to reflect maximal departmental variation in gender, age, educational background, clinical experience, and ethnicity. Recruitment of nurses and haematologists followed the patient participants, as nurse and haematologist participants were those with whom the patients had met on the day of observation. Nurses and haematologists were obligated to participate as part of the department’s research strategy but could choose to decline observation during the field study. The patients’ visits were pre-booked and patients visited either a haematologist, a nurse, or a haematologist followed by a nurse. The rationale for the interview approach was that the interviews conducted during the first and second studies were short and focused on the consultations observed (see Table 1). Subsequent interviews were undertaken to further elaborate on the research questions. The nurses at the outpatient clinic were well-acquainted with each other and volunteered to participate in the focus group interview, which resulted in a confident and dynamic discussion. The in-depth interviews with haematologists were conducted individually with volunteers, as the department reported that haematologists often decline to participate in group interviews out of ethical or professional obligations.

**Table 1:** Patients, nurses and haematologists participants included in the qualitative study

Patients						
	Gender	Age	Diagnosis	Intervention	Consulted by	Study phase
P1^a^	Male	74	Polycythemia vera	Control	N1	F1
P2	Female	73	Waldenström macroglobulinemia	Control	H2	F1
P3^a^	Male	85	Myelodysplastic Syndrome	PROMs available	H1	F1
P4	Female	71	Follicular lymphoma	Control	H3	F1
P5	Female	74	Myeloproliferative neoplasms	PROMs available	H4	F1
P6^a^	Male	68	Chronic lymphocytic leukemia	PROMs not available	N3 + H5	F1
P7^a^	Male	78	Myelodysplastic Syndrome	Control	H6	F1
P8^a^	Male	72	Follicular lymphoma	PROMs available	H7	F1
P9^a^	Male	71	Essential thrombocythemia	PROMs available	N4 + H8	F2
P10^a^	Male	75	Myelofibrosis	PROMs available	H6	F2
P11^a^	Male	74	Mantle cell lymphoma	PROMs not available	N7 + H9	F2
P12	Female	71	Waldenström macroglobulinemia	PROMs available	N6 + H10	F2
P13^a^	Male	76	Chronic lymphocytic leukemia	PROMs available	H11	F2
P14^a^	Female	77	Polycythemia vera	PROMs not available	H12	F2
P15	Male	83	Myelodysplastic Syndrome	PROMs available	N1	F2
P16^a^	Female	70	Follicular lymphoma	PROMs not available	H13	F2
Nurses						
	Gender	Experience as a nurse, years				Study phase
N1	Female	> 20 years				F1, F2, FG
N2	Female	> 15 years				F1, FG
N3	Female	> 10 years				F1
N4	Female	> 15 years				F2
N5	Female	> 10 years				FG
N6	Female	> 10 years				F2
N7	Female	< 5 years				F2
N8	Female	> 5 years				FG
N9	Female	> 10 years				FG (S)
Haematologists						
	Gender	Educational status/ Haematological experience				Study phase
H1	Male	Medical assistan t > 5 years				F1
H2	Male	Medical assistant > 5 years				F1
H3	Female	Medical assistant > 10 years				F1
H4	Male	Senior registrar				F1
H5	Male	Medical assistant > 15 years				F1
H6	Male	Medical assistant > 10 years				F1, F2
H7	Male	Medical assistant < 5 years				F1
H8	Male	Medical assistant > 10 years				F2, INT
H9	Female	Medical assistant				F2
H10	Male	Medical assistant > 10 years				F2
H11	Female	Senior registrar				F2, INT
H12	Female	Senior registrar				F2
H13	Male	Senior registrar				F2
H14	Male	Medical assistant < 5 years				INT

F1 refers to data from Field Study 1, F2 refers to data from Field Study 2, FG refers to data from Focus Group Interview and INT refers to data from the in-depth interviews. ^a^ Patients accompanied by a relative (S) on sick leave.

All participants agreed to the researcher being present during field observations. Participants are referred to by pseudonyms when transcripts are quoted. In the presentation of findings, ‘P’ followed by an individual number refers to a specific patient, ‘N’ followed by an individual number refers to a specific nurse, and ‘H’ followed by an individual number refers to a specific haematologist. F1 refers to data from Field Study 1, F2 refers to data from Field Study 2, INT refers to data from the in-depth interviews, and FG refers to data from the focus group interview.

### Procedure

**Fig. 1:**
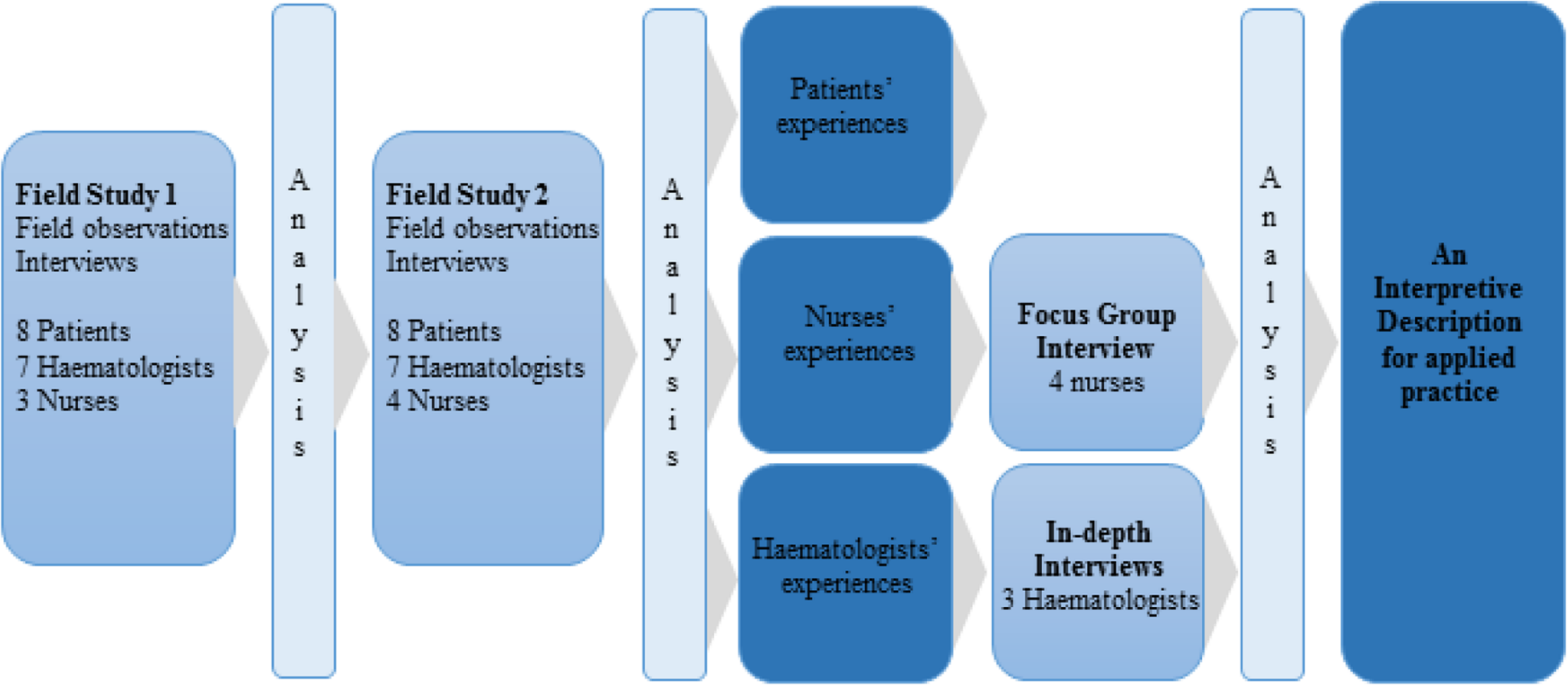
Illustration of concurrent multi-perspective data collection

Observations and interviews were conducted between March 2017 and December 2018 at a Danish university hospital. Observations took place in the natural social context of clinical practice. Inductive, concurrent, multi-perspective data collection and analysis was used for the three previous studies (See Fig. 1). Focused ethnography, including focused field observations and interviews, was applied to best explore the setting-specific, problem-focused and short-duration consultations between patients and haematologists or nurses (see Fig. 1). Field observations and interviews were planned for the three individual studies, and not specifically for eventual transverse data triangulation and analysis. Following patient consultations, the subsequent interviews were short and focused by necessity, as they were conducted between consultation appointments, while the in-depth interviews were allotted unlimited time. The short interviews aimed to investigate participants’ reflections and actions in the consultation observed, while the in-depth interviews aimed to explore participants’ general attitudes, rational basis, and experiences related to PROMs. A semi-structured interview guide (and related observation guide) provided guidance for each of the three studies, including descriptive, structural, and contrast questions (see Additional file 3) [32]. The interview guide covered thematic topics related to the users’ personal experiences, beliefs and knowledge on PROMs, and pragmatic considerations regarding the integration of PROMs in clinical practice. Observations during consultations lasted between 8 min and 6 h [30]; time variation occurred because some patients received a brief injection while others required longer-duration blood transfusions or intravenous medications. The interviews with patients, nurses and haematologists lasted 20 to 45 min. The focus group interview with the nurses lasted 1 hour [31] and the final in-depth interviews with haematologists lasted 17 to 47 min (see Fig. 1).

Observations, interviews, and the focus-group interview were conducted in Danish, audio recorded, and then manually transcribed and organized in NVivo PRO™ by the first author. Quotes were translated forward and backward by the first author from Danish into English after the analysis, according to quality assurance criteria for accuracy and correct usage of language in medical translation [39]. Final translations were agreed upon by all authors.

Interpretive Description does not prescribe a stepwise procedure for analysis: reducing qualitative research to a list of technical procedures is seen as overly prescriptive and may compromise the unique contributions of systematic and thoughtful qualitative research [40]. Instead, Interpretive Description focuses on broader concepts of quality in applied qualitative research to maintain sufficient rigor and ensure the academic credibility, including: epistemological integrity, representative credibility, analytic logic, interpretive authority, moral defensibility, disciplinary relevance, pragmatic obligation, contextual awareness, and probable truth [28]. The key to quality in Interpretive Description is primarily the internal logic of purpose, process and context that arrives at a coherent and convincing account [28].

### Data analysis

To generate an overall interpretive description for applied practice based on the three PROM user groups, further analysis was required [28]. The analysis process was inspired by nominal group technique^3^ and consisted of two distinct approaches to the total dataset [41]. First, the study results from the three user group perspectives were summarised, compared and discussed in the research group (see Table 2). This process sought to identify similarities and differences, add contextual structure, and interpret the findings. Second, the full dataset was reworked and codings previously excluded due to low relevance were grouped into themes for comprehending, synthesizing, theorizing, and re-contextualizing. This process was undertaken by the first author and discussed with the second and fifth author. Finally, the dataset was re-worked by the first author and agreed upon by all authors [38]. Disagreements were resolved by discussion, which led to insights that provided refining of themes. The labeling of themes was inspired by Habermas’ critical theory. For example, a theme related to structural similarities was found to be a consistent contextual factor for different user groups and their actions at the clinic, actions which may or may not be related to organizational regulations: such an observation relates to Habermas’ social ontology of the system, in this case the hospital organization.

**Table 2:** Qualifications and experiences of the research group

Author / Role	Professional Education/ Academic titles	Core Competencies and Experiences
1 / Insider Researcher	PhD, MScN, RN	Haematology nurse User experiences Implementation of PROMs Qualitative research Mixed methods Complex interventions
2 / Insider Researcher and Supervisor	PhD, MScN, MLP, RN	Qualitative research Applied nursing research Practice development in nursing Person-centred care Interpretive Description methodology
3 / Insider Researcher	MD, PhD Student	Haematology RCT-studies Implementation of PROMs
4 / Co-researcher and Supervisor	PhD, MD	General practitioner Evidence-based medicine Psychometrics Mixed methods Medical screening Overdiagnosis Development and validation of PROMs Multimorbidity Qualitative research
5 / Co-researcher and Main Supervisor	PhD, MScN, MA, RN	Qualitative research Mixed methods User experiences Capacity building in nursing research Nursing research Person-centred care Interpretive Description methodology

*Insider researcher: A researcher associated with the department under investigation.*

The integration of perspectives from the three user group datasets led to identification and categorisation of the data obtained from each group’s perspectives and combinations thereof. Deviant cases, if relevant to the current study’s research question, were discussed among the authors and integrated into the manuscript. Throughout the analysis process, a rigorous, comprehensive understanding was developed, leading to new themes.

## Results

Two overall themes emerged from the analysis: 1) “Structural similarities influence the adoption of PROMs,” and 2) “Different perspectives on the potential of PROMS.” The themes are described in detail below.

### Structural similarities influence the adoption of PROMs

The analytic process identified the theme “Structural similarities influence the adoption of PROMs,” which was explored and categorized into three subthemes: “The haematological agenda rules,” “Relationships are fundamental before adopting PROMs,” and “Structures hold the system.” These findings represent an assembled interpretation of transverse, contextual underpinnings related to PROMs.

#### The haematological agenda rules

The most prominent theme in the data was the ‘haematological agenda,’ which permeated clinical practice and patients’ everyday experiences. The haematological agenda represents the focus on and use of biomedical knowledge at a pathologic cellular level by haematologists and nurses to diagnose and treat haematological diseases. Haematological treatment focuses on cell status and moderation of cells with medical treatment, with results measurable via blood tests; in addition to tracking treatment results, blood tests are crucial because patients diagnosed with haematological diseases are continuously at risk of disease progression, which can include acute leukaemia. The haematological agenda focuses almost exclusively on biomedical outcomes, and does not focus on patient experiences or alleviation of secondary symptom burden or side effects. The haematological agenda acted as a barrier, as it overruled the potential use of PROMs:

When I am here, my physician knows that the first thing I want to know about is what my blood test shows … I know what it means if some of the blood values are suddenly red on the screen … Anyway, I am fine now, you never know if my blood turns back in haemolysis … I expect it all the time … (P6, F1)

When the patient is here, I have a very short time … I certainly have to find out about the status of the disease and then if medical treatment should be changed or adjusted … That is my speciality … (H1, F1)

I spend a lot of time reading patients’ electronic medical records because I am so scared to make any mistakes. What if I give the wrong treatment … The treatment is mainly why patients come to the outpatient clinic … (N2, FG)

#### Relationships are fundamental before adopting PROMs

Another similarity across patient, nurse and haematologist perspectives was the importance of relationships as fundamental to build trust: relationships were valued higher than application of PROMs. Many patients relied on the format of the consultations and the importance of meeting the same haematologist or nurse each time. As numerous patients expressed, “We know each other.” The haematologists reported that the relationship with the patient was more important than PROMs. Nurses mostly expressed that relationships and continuity were a necessity to work with PROMs:

The contact-person arrangement^4^ we nurses have in relation to the patients is definitely the most important … because it takes time to get to know each other … Talking with patients about these issues necessitates a good relation … When the relation is established, one can start to make a difference. (N8, FG)

The patients and I … We know each other. In most cases, I have been their haematologist for a very long time. So I know when something in worsening … And the patients may present what is on their mind to me … (H8, INT)

I always trust what my physician says … and I am very satisfied to see the same person every time I am here … Then we do not have to start all over every time … (P11, F2)

#### Structures hold the system

It was clear that although they worked in the same environment, nurses and haematologists had fragmented practices. This was expressed through their rationale of actions mainly determined by professional obligations, relying on the system:

This consultation was an overbooking as my calendar was full … But the patient has the right to get the result from the CT scan in decent time … That was the simple objective of her consultation. There is not so much to consider about the content … That is how it is. (H3, F1)

I do not understand the questions you ask [about PROMs] … I do not feel that they reflect my condition and my doctor for sure is not interested in this information about me. He always asks the same questions, those related to my disease … (P12, F2)

How long a time I have with the patient depends on which treatment the patient is having. So I might have a patient whose treatment takes about 10 minutes. Then I am only booked for half an hour, because I have to process the documentation as well … So even though the patient may have other needs, there is no time for it … (N7, F2)

Patients expected to receive information on their disease status from the haematologist while nurses provided their treatment. Haematologists were mostly restricted to the patient consultations according to specific aims, such as general follow-up or reporting test results. Such expectations and restrictions represent the structures and rationalities of action defined by the system, and were guided by nurses’ and haematologists’ professional obligations to the outpatient clinic.

### Different perspectives on the potential of PROMs

The theme “Different perspectives on the potential of PROMs” reflects transverse issues that influenced user perspectives on PROMs. The interpretation yielded three subthemes: “The choice of PROM instrument is of utmost importance,” “PROMs call for action,” and “Contradictory preferences.”

#### The choice of PROM instrument is of utmost importance

The specific PROM instrument influenced its use (or lack thereof) in the clinic, a finding that differed slightly depending on the user group. Information gained from the PROMs was seen as mostly irrelevant by patients and haematologists, while nurses expressed that the instrument yielded highly relevant information about patient conditions. Patients said the PROMs rarely reflected their condition, and haematologists viewed the PROM data as more relevant to general practitioners; some haematologists requested separate PROMs with specific relevance to haematological diseases and medical treatment. Nurses reported that the PROMs had potential, as they would like to initiate care using the new information; however, nurses had a limited ability to provide supportive care in the haematological outpatient clinic setting:

As nurses, the PROMs provide us with new information about patients’ conditions, which we do not get normally … Moreover, the information is in highly relevant areas [and] we could provide better supportive care … (N8, FG)

The information provided through PROMs could be for any patient diagnosed with cancer … I would rather like to know about specific haematological issues … such as if the patient has night sweats … or if the patient has noticed any abnormal enlargements around the lymph nodes … (H7, F1)

Well the questions were fine … I did not have any problems answering … But at some point, I felt you were asking me as if I was an intensive care patient … Like, am I really so sick? (P6, F1)

Even though nurses viewed the PROM data as relevant, the data also represented a potential bias risk. If allowed time to address needs identified by a patient’s PROM, nurses might prioritize supportive care related to the highest-rated outcome; however, if the PROM instrument is not designed to initiate care, higher ratings could be misleading. Nurses run the risk of failing to address critical patient issues if a PROM does not accurately reflect the patient’s condition, leading to potentially harmful situations resulting from data-driven decisions.

Another finding was that individual professionals assessed the relevance of PROMs, and their opinions acted as a barrier to use of the PROM data.

#### PROMs call for action

A shared finding among patients, nurses and haematologists was that PROMs call for action by clinicians and that the PROMs were intended to initiate communication and action, not simply to gather data. However, there was a lack of action on the PROM data. Nurses and haematologists had no procedure to follow for implementation of PROM findings, and usage of PROMs during patient consultations was not monitored. For the patients, completion of PROMs did not lead to asking haematologists or nurses about the PROM findings:

… I did think it was a bit strange … When I answered the questions … I was asked whether I felt pain … and I answered that I did … I wonder, when I write that I am in pain and that I hardly sleep … why does no one inquire into those questions? Or maybe that is not the purpose? (P13, F2)

In my view … These PROMs open up a mountain of potential communication between the patient and I … which I am not interested in … Those potential problems are not my duty, so why waste our time? (H6, F2)

It is sad to say … but when I am with a patient I do not ask all these questions about psychosocial issues … I am afraid to open up issues in relation to the patient which I am not able to fully support because my time is limited … And next time the patient is probably going to meet some other nurse here … (N8, FG)^5^

#### Contradictory preferences

Although they all believed in the potential of PROMs, patients, nurses and haematologists had different preferences regarding relevance of PROMs, which often depended on the purpose of a patient consultation. Nurses expressed that they were trained to provide supportive care and wanted to do so, as this is the core of the nursing discipline; however, their work was organized in such a way that they were required to prioritize mandatory medical treatment, such as infusion of chemotherapy. The nurses had neither the time nor the latitude to pursue supportive care in other areas. Haematologists viewed the PROMs as irrelevant; their preference would have been to receive data on disease status and adjustment of treatment. Applying another PROM instrument would probably not increase the haematologists’ use of PROM data, as they did not see supportive care as a core responsibility. For the patients, the questions were not accurate for their situation and thus often irrelevant.

Overall, nurses and haematologists had contradictory preferences regarding PROMs, and neither group claimed responsibility for initiating supportive care related to issues raised in the PROM instrument. Some patients voiced an awareness of their own unmet needs due to the questions in the PROM instrument. Other patients could not identify with the questions and wished to abstain from completing PROMs, or asked to write free-text answers. Nurses faced a professional dilemma, as an inability to provide care related to the PROM data conflicted with their professional and ethical obligations. Haematologists relied upon the haematological agenda and viewed the PROM data as identifying needs best fulfilled by others, specifically general practitioners. These actions and viewpoints complicated the implementation of PROMs, representing both facilitators and barriers.

In this case, I tried to explore the PROM information … However, you saw how the consultation turned out to deal with many issues not related to patient’s disease or treatment … Therefore, we did not have time to talk about the patient’s treatment, which is why he was here … That is a huge problem! (H1, F1)

Of course I would like my doctor to care about my constipation … but I rather prefer that he takes care of my myelofibrosis … if you ask me to prioritize … eventually, I can solve the constipation myself. (P13, F2)

So the patient was here for venesection and I did not notice the PROMs. I actually feel bad now that I see that the patient stated some issues which I should have asked about … My focus was simply to get going on the venesection … . He [the patient] said that sometimes the nurses were challenged finding his veins, and that was my concern at that point. (N4, F2)

## Discussion

Examining data from three qualitative studies of user experiences with PROMs in a haematological outpatient clinic, this study aimed to investigate the barriers and opportunities for PROMs in the future, specific to this setting.

In the present study, two overall themes were identified: “Structural similarities influence the adoption of PROMs” and “Different perspectives on the potential of PROMs.” The first theme comprises the interconnected haematological agenda of clinical practice. Users identified a shared focus on the haematological agenda as the core obligation, a fundamental need for relational work, and a recognition of structures and system rationality. The second overall theme identified the need for the PROM instrument to match the construct: successful application of PROMs requires a chain of actions, and nurses’ and haematologists’ preferences related to PROMs diverged.

Overall, we found that PROMs were perceived very differently by patients, nurses and haematologists, sometimes in ways that were inconsistent with the department’s declared values of person-centered care and interactional practice [43]. These contradictory perceptions of the PROM instrument may explain why the PROMs were not fully adopted in clinical practice. Our study also identified cases where patients reported symptoms in their PROMs, such as constipation, which were not addressed by the clinicians, either for treatment or referral to a general practitioner [32]. Failure to address items reported in the PROMs left some patients with untreated symptoms or conditions that adversely influenced their health and health-related quality of life.

One study revealed that PROMs can have unintended consequences: PROMs focus on precisely defined, measurable aspects of quality of life, which can lead to a narrow focus on quantifiable data, thus disregarding vague complaints, social diagnoses, and physical manifestations of underlying clinical problems [44]. That same study also found that operationalizing PROMs by requiring or incentivizing adoption may result in unanticipated and potentially harmful effects in busy practice settings [44]. Our study supports this finding, as in the case where a haematologist did not address a patient’s constipation symptoms identified in PROMs [45], which might be harmful [10, 46]. Ethically, there is an obligation to inquire why healthcare professionals would fail to act when learning of a patient’s condition. Haematologists perceived that their primary duty was to provide patients with their disease status and inform them about treatment adjustments [32]. However, this haematology department prescribed specific guidelines for follow-up care [42], such as recommending treatment and supportive care for incidental findings.

A recent review exploring PROMs as a support for clinician-patient communication further supports our findings [5]. Greenhalgh and colleagues found that the way clinicians used PROMs was shaped by their relationship with patients, and by professional roles and boundaries; clinicians’ adoption of PROMs was influenced by the process of building relationships with patients [5]. Moreover, the authors concluded that PROMs do not provide information on the *causes* of poor care: providers need to integrate and further interpret PROM data and other outcomes in the context of the total information available for an individual patient [5]. Another study found that the use of PROMs can improve physician satisfaction, enhance physician-patient relationships, and enable crucial conversations [47]. These findings confirm that the implementation of PROMs will be affected by the setting, which is in turn crucial to how PROMs will be perceived by potential users.

Contrary to our results on patient perspectives, a recent PROM study within the haematological field found that PROMs could assess treatment effects on health-related quality of life from the patients´ perspective [48]. However, the study struggled to achieve patient compliance with questionnaire completion and the researchers expended great effort to remind patients about completion, thus resulting in a high response rate. This could represent an ethical issue or affect validity, since lack of completion could be interpreted as an expression of e.g. dissatisfaction, and an indicator in itself of importance. A patient might also lack the necessary resources to achieve compliance; the content might be hard to understand, irrelevant or incomprehensive. If such problems are plausible reasons for low completion rates, forcing patients to complete PROMs can invalidate the outcome [49, 50]. This further highlights the fact that the adoption of PROMs is highly dependent on the specific PROM tool and its setting, design, and study setup [51, 52].

In previous publications [30–32] we have discussed the individual perspectives of patients, nurses and haematologists in relation to Habermas’ critical theory [36, 37], especially exploring the system versus lifeworld perspective [30–32]. Taking this one step further, critical theory may help explain how the three user group perspectives apparently manage to function, unproblematically, side-by-side in everyday clinical practice. The haematological agenda’s biomedical discourse, dominant across all three user group perspectives, represents what Habermas refers to as intersubjectivity, or a norm [36, 37]. The rationale for patients, nurses and haematologists in this study was built on what Habermas terms cognitive-instrumental reasoning [36], meaning that the biomedical aspects, objectivity and efficiency dominated patient consultations.

The patients, nurses and haematologists in the present study saw the PROMs as a potential route to improved care, which Habermas would call communicative rationality, consisting of communication and interaction. This is in line with nursing epistemology, which according to Thorne is characterized by moral-practical reasoning, focusing on the positive and pragmatic, and on justice and influencing society [53]. However, despite an optimistic attitude toward PROMs, the nurses in this study did not use the PROM data and explained that lack of time required a focus on mandatory tasks related to treatment, control and documentation. From a critical theory perspective, this represents a communicative reality and can be seen as an example of how the system’s values have become the norm in modern health care, limiting human creativity and freedom to act and develop [36].

Studies have shown that lack of ability to conduct core professional duties can lead to health professionals leaving their profession [54–56]. This study should therefore provoke further discussion among policy makers and healthcare managers in clinical practice, especially since most of the world currently struggles with a serious shortage of healthcare personnel. One suggestion for how to advance the healthcare system as an attractive place to work is to safeguard the structures around which services are expected to be performed and documented, including extending services from the strictly biomedical to the holistic.

Relating to the perspectives of patients, chronically ill patients accepted and were socialized into the haematological agenda via their long-term connections to the outpatient clinic and overall dependence on the system. They were accustomed to being defined and evaluated by biomedical investigation via clinical lab tests. The haematological agenda was therefore perceived as more important than PROMs, as potential disease progression was a threat to patients’ lives. Furthermore, the objective for the PROMs—to develop a holistic assessment of patients’ conditions—did not fit the clinical context, providing information beyond the haematological agenda. The nurses and haematologists did not intervene using PROM data and the patients did not question that, at least not during the consultations. Recent studies within oncology suggest one answer: patients may feel emotionally supported even without explicit emotional talk, because they view doctors as experts who have the knowledge and authority to treat them [57].

A final point for discussion in our study findings is the department’s approach to PROM implementation, which in retrospect may have been insufficient. Within the multimethod project, implementation was defined as a complex and sometimes unplanned process:

Implementation is part of a diffusion-dissemination-implementation continuum: diffusion is the passive, untargeted and unplanned spread of new practices; dissemination is the active spread of new practices to the target audience using planned strategies; and implementation is the process of putting to use or integrating new practices within a setting [58, 59].

The implementation of PROMs in this study can be characterized as an early-phase dissemination-implementation strategy, as it did not prescribe concrete actions for clinicians to perform, e.g. how to react to patients’ PROM responses, or thresholds for action based on the sum scores of the scales. Considering implementation as “the process of putting to use or integrating new practices within a setting” [59], it is questionable whether the PROMs in this study can truly be said to be implemented, since we found they were hardly used. Implementation has been described as a complex process of stages which cannot be skipped, and translating knowledge or research into clinical practice is complex [60]. Unless knowledge is put into action, the potential benefits of PROMs cannot be realized. Consistent with interpretive description’s focus on context, the findings of the present study explore both suitable and inconvenient aspects of implementation. The next step should be to create alternatives for clinical practice with PROMs that are better, stronger, and more critically informed in response to the facilitators and barriers identified [28].

## Strengths and limitations

The three user group perspectives were examined together, ensuring coherence between the separate studies and allowing for a cross-group analysis of the unsuccessful implementation of PROMs. Specific study limitations related to the experiences of patients, nurses and haematologists are described separately within previously published papers [30–32]. In the multimethod project, PROMs were intended to be incorporated according to the haematology department’s instructions on supportive care. This approach relied upon the rationale that haematologists and nurses would take action when receiving information and that further bureaucracy (e.g. monitoring of PROM usage) would be an unnecessary inconvenience. This assumption may have acted as a limitation and shaped our findings about nurses and haematologists not using PROMs. The current study identifies knowledge about these limitations and barriers as well as potential opportunities for future use of PROMs in clinical practice.

Another study limitation was that the patients recruited for the qualitative study were a selected group: they were already included in the larger multimethod project, which could explain why all the patients viewed PROMs optimistically. In total, 220 (54%) of the 404 eligible patients consented to participate in the multimethod project, an inclusion rate which could indicate participation bias, as a large proportion of these patients did not find reason to participate [61]. Analysis of potential socio-demographic differences and reasons for nonparticipation in the multimethod project could have provided important information about participation bias and barriers to future PROM interventions.

An additional limiting concern for this study is the patients’ relatives, whose perspectives were not included in the multimethod project. Commonly, relatives act as a resource for patients in the healthcare system [12]. For example, once during the study, the first author became aware that a patient had not completed the PROMs; the patient’s spouse had completed it, answering questions on behalf of the patient. Currently, there is no method for the incorporation of relatives in PROMs, but most patients are encouraged to be accompanied by relatives to the hospital. Therefore, involvement of relatives should be considered in future PROM integration and research.

Another potential limitation is the overall project set-up. In the best scenario, the first author should not have been involved in the administration of the multimethod RCT study. Introducing PROMs, teaching colleagues how to use PROMs, and investigating and evaluating their use might not have been optimum; the first author’s different roles and responsibilities could represent an intellectual conflict of interest, with inadequate independence from (and thus ability to critique) the RCT study. This set-up was, however, a pragmatic solution for the multimethod project, and at the same time represented the qualifying condition which allowed the first author to investigate the field. The first author was also formerly employed at the haematological outpatient clinic where the study took place [28]. This role facilitated access to the field and welcoming from the staff, but it also necessitated a specific focus on bias, presumptions, and prejudices [62]. Critical reflections on the development and role of the researcher’s subjectivity have been described in greater depth elsewhere [34]. Evaluative criteria for reflexivity and subjectivity were applied as described within the procedure section of this study, in terms of how the research process might have influenced or limited the findings [63]. Credibility was strived for, using transparency to reinforce trustworthiness and accuracy in the process and findings [28].

These limitations infer that this study’s findings are highly related to the current study and not easily transferable to other settings. The study’s implications are specific to the particular study and practice, though its findings may add relatable knowledge to other haematological practices or outpatient clinics.

## Conclusion

When PROMs were implemented in a haematological outpatient clinic, all user groups demonstrated an unquestioned commitment to the haematological agenda as common sense and to the norms of the system, and thus did not use the PROMs. We found that structural similarities influenced the non-adoption of PROMs and the different perspectives on the potential use of PROMs. Nurses and haematologists expressed different preferences and objectives for the use of PROMs in clinical practice, objectives which were not compatible with their clinical practice in context.

### Practice implications

1. When applying PROMs in clinical practice, an objective should be clearly defined: Do the PROM scores represent a threshold for action, and if so, what actions should be taken?
2. This study did not assess content validity but the findings indicate that the PROMs applied were not appropriate for the specific setting and patient group [64]. Future PROMs should be carefully selected in collaboration with all potential users to ensure high content validity.
3. If PROMs are intended to be prioritized in a system context, the system should require and facilitate the use of PROMs. PROMs should be introduced with an implementation strategy addressing healthcare professionals’ responsibility to intervene and address patient needs.

### Implications for research

Future research on PROMs should address context issues, moving towards a feasible and convenient model or framework.

## Supplementary information

**Additional file 1 **Adapted as original from the publication: Thestrup Hansen, S., Kjerholt, M., Friis Christensen, S., Brodersen, J., & Hølge-Hazelton, B. (2020). “I Am Sure That They Use My PROM Data for Something Important.” A Qualitative Study About Patients’ Experiences From a Hematologic Outpatient Clinic. *Cancer nursing*, *43*(5), E273–E282

**Additional file 2 **Adapted as original from the publication: Thestrup Hansen, S., Kjerholt, M., Friis Christensen, S., Brodersen, J., & Hølge-Hazelton, B. (2020). “I Am Sure That They Use My PROM Data for Something Important.” A Qualitative Study About Patients’ Experiences From a Hematologic Outpatient Clinic. *Cancer nursing*, *43*(5), E273–E282.

**Additional file 3 **Adapted as original from the publication: Thestrup Hansen, S., Kjerholt, M., Friis Christensen, S., Brodersen, J., & Hølge-Hazelton, B. (2020). Nurses’ Experiences When Introducing Patient-Reported Outcome Measures in an Outpatient Clinic: An Interpretive Description Study. *Cancer Nursing*. https://doi.org/10.1097/NCC.0000000000000808

## Data Availability

The datasets generated and/or analysed during the current study are not publicly available but are available from the corresponding author upon reasonable request.

## Abbreviations

ID: Interpretive Description
PROM: Patient Reported Outcome Measures
RCT: Randomized Controlled Trial

## Footnotes

1 Interpretive Description is a qualitative research methodology for applied practice [28]

2 Jürgen Habermas is a German philosopher and sociologist in the tradition of critical theory and pragmatism [29].

3 Nominal group technique is a structured research group interaction which provides the participants an opportunity to have their voices heard and opinions considered by other members [41]

4 According to the department’s guideline for follow-up care [42], patients are assigned a permanent primary haematologist and a nurse at the outpatient clinic.

5 Patients are assigned a permanent primary haematologist at the outpatient clinic while no primary nurse contact arrangement is established.
