# Supplementary material for: User experiences on implementation of patient reported outcome measures (PROMs) in a Haematological outpatient clinic
Source: J Patient Rep Outcomes. 2020 Oct 28;4:87. doi: 10.1186/s41687-020-00256-z (PMC7593370; doi:10.1186/s41687-020-00256-z)

**Additional file 1**. Adapted as original from the publication:

Thestrup Hansen, S., Kjerholt, M., Friis Christensen, S., Brodersen, J., & Hølge-Hazelton, B. (2020). "I Am Sure That They Use My PROM Data for Something Important." A Qualitative Study About Patients' Experiences From a Hematologic Outpatient Clinic. *Cancer nursing*, *43*(5), E273–E282.
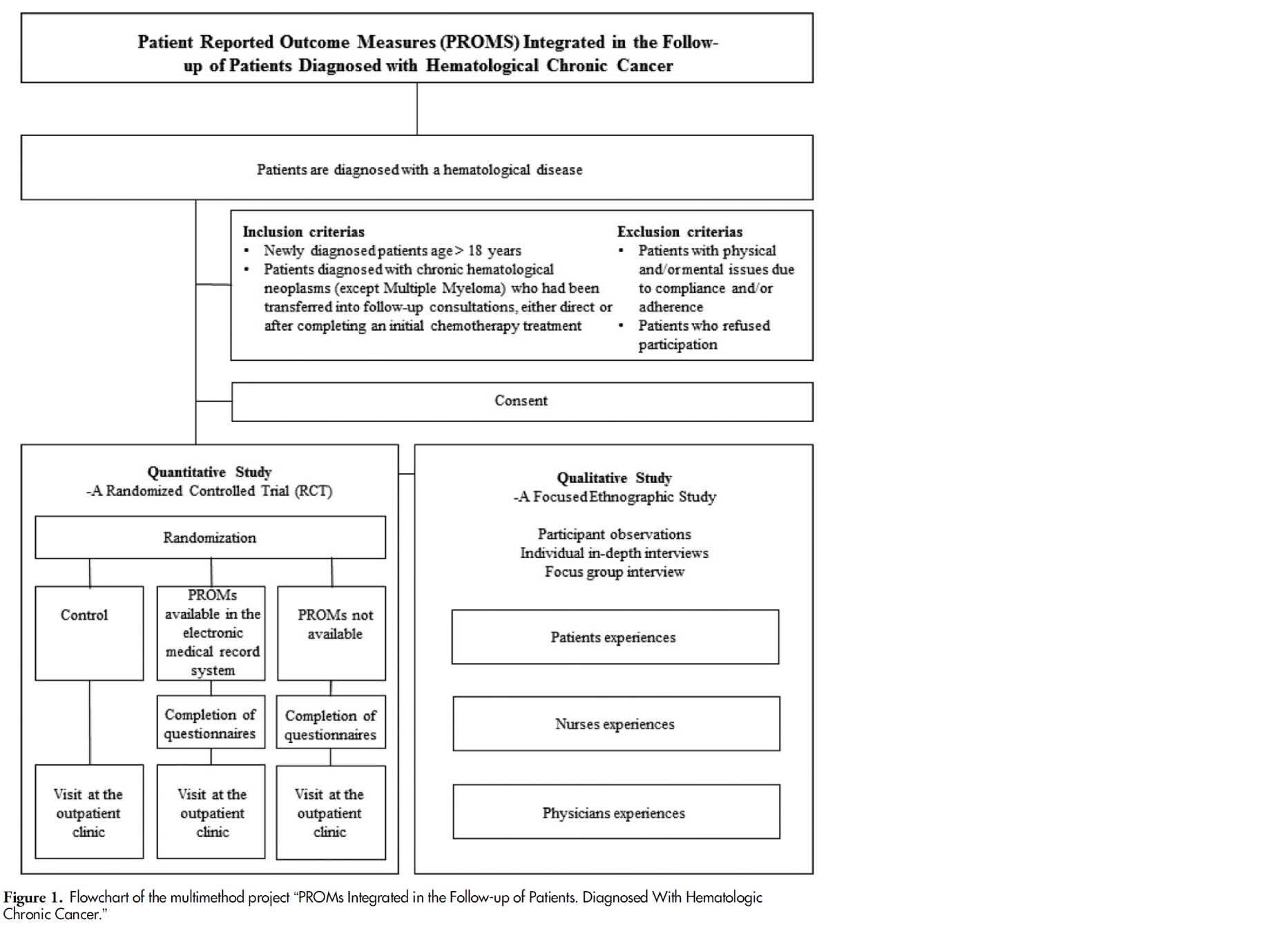

Supplement: Supplementary file 1 — Additional file 1 Adapted as original from the publication: Thestrup Hansen, S., Kjerholt, M., Friis Christensen, S., Brodersen, J., & Hølge-Hazelton, B. (2020). “I Am Sure That They Use My PROM Data for Something Important.” A Qualitative Study About Patients’ Experiences From a Hematologic Outpatient Clinic. Cancer nursing, 43(5), E273–E282 [file 41687_2020_256_MOESM1_ESM.docx]
