# Supplementary material for: User experiences on implementation of patient reported outcome measures (PROMs) in a Haematological outpatient clinic
Source: J Patient Rep Outcomes. 2020 Oct 28;4:87. doi: 10.1186/s41687-020-00256-z (PMC7593370; doi:10.1186/s41687-020-00256-z)

***Additional file 3.*** *Adapted as original from the publication:*

Thestrup Hansen, S., Kjerholt, M., Friis Christensen, S., Brodersen, J., & Hølge-Hazelton, B. (2020). Nurses' Experiences When Introducing Patient-Reported Outcome Measures in an Outpatient Clinic: An Interpretive Description Study. Cancer Nursing. <https://doi.org/10.1097/NCC.0000000000000808>


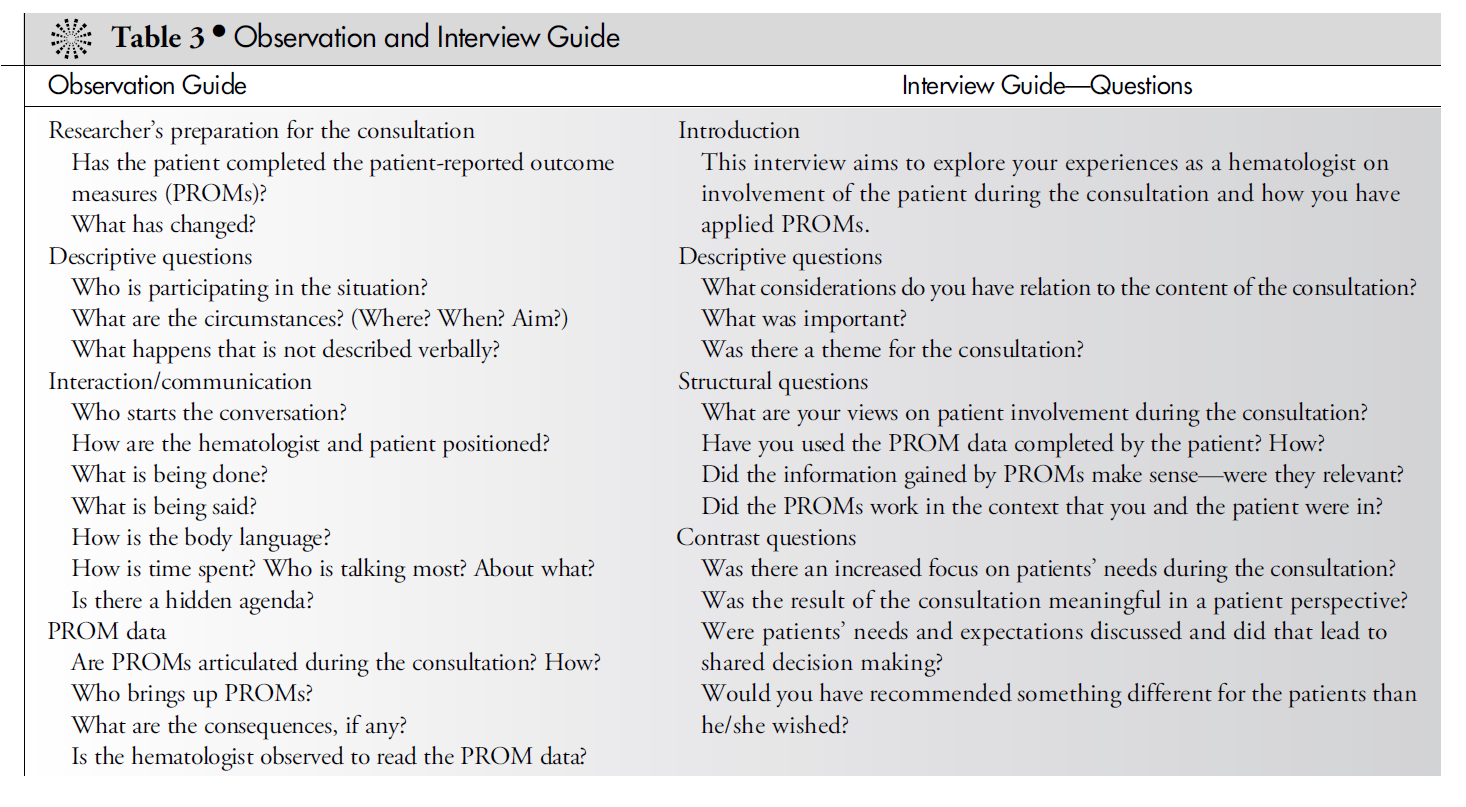

Supplement: Supplementary file 3 — Additional file 3 Adapted as original from the publication: Thestrup Hansen, S., Kjerholt, M., Friis Christensen, S., Brodersen, J., & Hølge-Hazelton, B. (2020). Nurses’ Experiences When Introducing Patient-Reported Outcome Measures in an Outpatient Clinic: An Interpretive Description Study. Cancer Nursing. https://doi.org/10.1097/NCC.0000000000000808 [file 41687_2020_256_MOESM3_ESM.docx]
